# Silver Sutures in Surgery

**Published:** 1858-04

**Authors:** 


### Silver Sutures in Surgery.

We have received the "Anniversary Discourse," before the New York Academy of Medicine, delivered on the 18th of November, 1857, by J. MARION SIMS, M. D., Surgeon to the Woman's Hospital. Published by order of the Academy.

This is a pamphlet of 79 pages, with 22 illustrations accompanying it, from original drawings by Dr. THOMAS ADDIS EMMET, the accomplished Assistant Surgeon to the Woman's Hospital, and, in addition, contains a catalogue of the Officers and Corresponding and Resident Fellows of the New York Academy of Medicine.

Without entering into the merits of any controversy which may exist between the author of this discourse and any other Medical gentleman, we feel constrained to say that we consider that a large debt of gratitude is due the eminent gentleman, not only from those who may be the unfortunate subjects of vesico-vaginal fistula, which has been so successfully treated by his method—and as the result only of the most untiring perseverance, under most discouraging circumstances—but from the whole Medical profession. We have been deeply interested in the history and progress of the laborious and persevering efforts which have finally resulted in one of the greatest achievements known to Surgical science, and covered the principal actor in its accomplishment with imperishable renown. The author seems to be fully sensible of the delicacy of the position which he occupied in the presentation of such a subject upon such an occasion, but we have no hesitation in saying that he was fully justified under the circumstances in thus placing upon record his claim and agency in this great discovery. Whether, in the ardor and enthusiasm of a devotee, he does not in some degree over-estimate the "Silver Suture" in general Surgery, we are not exactly prepared to say, but we presume that there is only one opinion as to the actual value of its substitution for silk, and the great merit which unquestionably attaches to his labors in connection with this subject, whatever modification or improvements have been or may be made in reference to it, and we fully agree, in conclusion, with the sentiment of the following extract from his address. He remarks:

"While I know that posterity will do me full justice, I do not even fear the verdict of my contemporaries, when the whole of the facts and their philosophy are laid plainly before a just and discriminating profession. But, Sir, if you wish to offer a premium for the encouragement of secret remedies, rob me of my well earned claims, as the discoverer and propagator of a great principle that shall live as long as surgery is cultivated as a science, or practised as an art;—and, if you are particularl y desi rous to drive your young men ambitious of honorable distinction, from their high resolves to elevate and ennoble our calling, show t em that you are ever ready to thus endorse any attempt to detract from th e meed of their self-sacrificing efforts.

An honorable sensitiveness on the score of professional claims and personal rights is natural, and comes home to every man, whether his reputation be already established, or merely prospective.—And no one, Mr. President, * is more capable of fully appreciating this than your honored self, whose brilliant surgical achievements have so often been the object of envy and detraction."

---

* Dr. Mott.

